# Genome-wide identification of DNA-binding with one finger transcription factor genes in Chinese chestnut and their response to abiotic stress

**DOI:** 10.3389/fpls.2025.1711429

**Published:** 2025-12-04

**Authors:** XiuRong Xu, Xibing Jiang, Shiming Cheng

**Affiliations:** 1 Zhejiang Academy of Forestry, Hangzhou, Zhejiang, China; 2 Research Institute of Subtropical Forestry, Chinese Academy of Forestry, Hangzhou, China

**Keywords:** DOF, gene family, identification, expression analysis, stress response

## Abstract

DNA-binding with One Finger (Dof) proteins are unique single zinc finger transcription factors that play important roles in plant growth, development, and abiotic stress responses. However, the *Dof* genes in Chinese chestnut (*Castanea mollissima*) have not been studied yet. The transcription factor family in the Chinese chestnut genome was identified and analyzed using bioinformatics. The analysis results revealed that a total of 25 *CmDof* genes (*CmDof1* – *CmDof25*) were identified in Chinese chestnut. Furthermore, we assessed the physicochemical properties, phylogeny, gene structures, *cis*-regulatory elements (CREs), and expression profiles. The 25 *CmDof* genes were categorized into five subfamilies according to the phylogeny analysis. Analysis of *cis*-acting elements revealed that the promoter region of the *Dof* gene in Chinese chestnut contains light regulation, plant growth and development, plant hormones, and stress-response elements to adapt to environmental changes. The RNA-seq data analysis indicated the potential roles of *CmDof* genes in the response to temperature stress, drought stress, and shade stress. Additionally, The expression levels of nine *CmDof* genes showed significant differences in their response to the three abiotic stresses, confirmed by RT-qPCR assays. Collectively, these data lay a foundation for further functional explorations of *CmDof* genes, especially concerning the possible application of multiple *CmDof* genes in breeding tolerant plants.

## 1 Introduction

The Dof (DNA binding with one finger) gene family is a plant-specific class of transcription factors that belongs to the single zinc finger protein superfamily. It participates in regulating physiological and biochemical processes, such as plant tissue differentiation, seed germination, plant stress resistance, and metabolism. Dof proteins are typically composed of 200–400 amino acid residues (aa), including two main structural domains: a conserved DNA-binding domain at the N-terminus and a transcriptional regulatory domain at the C-terminus. The N-terminus contains a highly conserved Dof domain composed of 50–52 amino acid residues (Cominelli et al., 2011). The Dof domain contains a C2-C2 type zinc finger structure, which can specifically recognize *cis*-regulatory elements with the core sequence AAAG (Yanagisawa and Schmidt, 1999; Yanagisawa et al., 2002a; Umemura et al., 2004). The C-terminal protein sequence is more variable than the N-terminal domain, which is one of the reasons why Dof transcription factors family in plants have diverse functions.

Recent studies have shown that the Dof transcription factors could participate in plant growth, organ development, and responses to various environmental stressors (Yanagisawa and Izui, 1993; Washio, 2003; Wang et al., 2021; Wang et al., 2020). The first *Dof* gene discovered in plants was *ZmMNB1*, which participates in the regulation of C4 plant photosynthesis by binding to the AAGG sequence on the MNF1 promoter (Corrales et al., 2014). Thereafter, many plant *Dof* genes have been identified. For example, 36, 30, 46,and 26 *Dof* family members have been identified in *Arabidopsis* (Yanagisawa, 2002), rice (Lijavetzky et al., 2003), maize (Chen and Cao, 2015), and pitaya (Alam et al., 2024a), respectively. *DAG1* (Dof affecting germination 1) and *DAG2* (Dof affecting germination 2) in *Arabidopsis* are Dof transcription factors that regulate the expression of genes related to seed germination (Gualberti et al., 2002). In addition, *OBP1* can control the expression of defense genes in *Arabidopsis* (Chen et al., 1996). Most *PheDof* genes in bamboo are involved in the responses to drought, low temperatures, and salt (Wang et al., 2016). The expression levels of *SlCDF1* and *SlCDF3* in the photoperiod response of tomatoes were the highest at the beginning of the photoperiod, whereas the expression levels of *SlCDF2*, *SlCDF4*, and *SlCDF5* peaked at night (Cai et al., 2013). However, the function of the *Dof* gene in Chinese chestnut remains unknown.

Evidently, the vital roles of the *Dof* genes in plant growth and development, metabolic regulation, and response to stress factors of plants have been extensively demonstrated. However, the function of the *Dof* gene in Chinese chestnut (*Castanea mollissima*) has not been explored in research. *Castanea mollissima* belongs to the genus *Castanea* of the *Fagaceae* family. The fruit is rich in nutrients and contains abundant starch, protein, fats, vitamins, and other nutrients (Zhang et al., 2023, 2023). and is therefore known as the “King of Dry Fruits”. Chinese chestnut has been regarded as an important ecological and economic tree species due to its rich nutrition, disease resistance and drought resistance. Chinese chestnuts are cultivated in the southern and northern regions of China. According to the Food and Agriculture Organization of the United Nations (FAO)(http://www.fao.org/home/en/), the nut yield of the Chinese chestnut in China is approximately 1.6 million tons, accounting for 83.3% of global production in 2020. However, there are few reports on the gene families in Chinese chestnut associated with its growth, development, and stress resistance, which undoubtedly limits our understanding of this miraculous plant.

This study employed bioinformatic approaches to identify the members of the Chinese chestnut *Dof* gene family and analyzed their chromosomal locations, gene structures, physicochemical properties, evolutionary relationships, and promoter elements. Additionally, the expression patterns of *CmDof* genes in leaves were examined under shade, high-temperature, and low-temperature stresses. Finally, qRT-PCR verification was performed to support further investigation of the functions of *Dof* family members, their response to environmental factors, and to provide molecular resources for breeding new Chinese chestnut varieties.

## 2 Materials and methods

### 2.1 Identification of *Dof* gene members in Chinese chestnut

The genome and protein files of Chinese chestnut were downloaded from the National Genomics Database of China (https://ngdc.cncb.ac.cn/gwh); the genome and protein files of *Arabidopsis thaliana* and *Oryza sativa* were downloaded from the Ensembl Plants database (https://plants.ensembl.org/); the genome and protein files of Japanese Chinese chestnut (*Castanea crenata*) were downloaded from the Plant Genome Portal database (https://plantgarden.jp/en/index); the genome and protein files of American Chinese chestnut (*Castanea dentata*) were downloaded from the Phytozome13 database (https://phytozome-next.jgi.doe.gov/info/cdentata_v1_1/); the Dof domain file with the number PF03083 was downloaded from the Pfam database (http://pfam-legacy.xfam.org/) and compared using HMMER software to obtain potential members of the Dof gene family in each species. Finally, the potential protein sequences were uploaded to the InterProScan database (https://www.ebi.ac.uk/interpro/result/interprosca/), which integrates protein domain annotation databases, such as Pfam, CDD, SMART, and PROSITE. These databases accurately identify the gene family of an unknown protein and confirm the true members containing the Dof domain.

### 2.2 Construction of the phylogenetic tree of the *Dof* gene family and determination of homologous genes

The identified *Dof* gene family members were compared using MAFFT software (Rozewicki et al., 2019), and a phylogenetic tree was constructed by MEGA 7.0 software using the maximum likelihood method in FastTree software (Price et al., 2010). Homologous genes in the *Dof* gene family were identified using the OrthoFinder program with an E-value of 1e-2 and a threshold of 1.5 (Emms and Kelly, 2019). The branches and homologous groups were displayed using the Chiplot software (Xie et al., 2023).

### 2.3 Chromosome localization and protein physicochemical properties analysis of *CmDof* gene family

The chromosomal positions of each *CmDof* gene were identified, and a chromosome distribution map of the *CmDof* gene family was drawn using TB tools (Chen et al., 2023). The CmDof protein sequences were uploaded to the Expasy ProtParam website (https://web.expasy.org/protparam/) to analyze the physicochemical properties of each family member. Finally, the CmDof protein sequences were uploaded to the BUSCA website (https://busca.biocomp.unibo.it/) to predict the specific location of each family member’s protein function in the cell.

### 2.4 Motif, conserved domains, and gene structure analysis of *CmDof* gene family

The MEME website (https://meme-suite.org/) was used to analyze the motifs of *CmDof* gene family protein sequences. Ten conserved motif sequences were obtained, and these conserved motif sequences were uploaded to the InterProscan database for annotation analysis. The conserved domains of *CmDof* gene family members were also analyzed using the Interproscan database. The gene structure of *CmDof* gene family members was analyzed using TBtools, and the motifs, conserved domains, and gene structures were displayed using TBtools (Chen et al., 2023).

### 2.5 Analysis of *cis*-regulatory elements of *CmDof* gene family

The 2000 bp promoter sequence before the ATG start codon of the *CmDof* gene family members was extracted using TBtools and uploaded to the PlantCARE database (http://bioinformatics.psb.ugent.be/webtools/plant-care/) for *cis*-acting element prediction. Finally, *cis*-regulatory elements were displayed using TB tools (Chen et al., 2023).

### 2.6 Analysis of gene duplication types of *CmDof* gene family

Collinearity analysis of the Chinese chestnut genome was performed using the MCScanX software (Wang et al., 2012). Based on the results of the collinearity analysis, the gene duplication types of the entire genome were analyzed using the DupGenFinder program, and the duplication types of the *CmDof* gene family members were obtained (Qiao et al., 2019). The duplication types of the *CmDof* gene family members in the other eight species(*Arabidopsis thaliana*, *Oryza sativa*, *Vitis vinifera*, Zea mays, *Quercus dentata*, *Castanopsis tibetana*, *Castanea dentata*, and *Castanea crenata*) were obtained in the same way. Finally, the non-synonymous substitution rate/synonymous substitution rate of the gene pairs with different duplication types of the *CmDof* gene family were calculated using TBtools software (Chen et al., 2023).

### 2.7 Expression pattern analysis of *CmDof* gene family during fruit ripening

Transcriptional data for Chinese chestnut fruit on the days70, 82, and 94 after flowering (SRP198418/PRJNA540079) were obtained from NCBI (Li et al., 2021b)using an Illumina sequencing platform. The reads were aligned to the Chinese chestnut ‘N11-1’ reference genome. Finally, transcripts per kilobase of exon model per million mapped reads (TPM) were used as indicators of gene expression.

### 2.8 Plant materials and RT-qPCR validation

To investigate differences in phenotypes, transcriptomes, and expression levels of chestnut leaves under varying abiotic intensities, we tested two-year-old seedlings exhibiting consistent growth potential under standard water and fertilizer conditions. These seedlings were subjected to shading treatment using black shading nets. Four shading intensity treatments (0%, 50%, 75%, and 95%) were employed in this experiment. After ten days of shading treatment, the third leaf from the top of the trees was collected, rapidly frozen in liquid nitrogen, and stored at –80°C. The trees were subjected to high-temperature treatment at 45°C, and leaf samples were collected after 4, 8, and 12 h. Additionally, they were subjected to low-temperature treatment at −15°C, and leaf samples were collected after 5, 10, and 15 h, respectively. Trees grown at 25°C were used as the control. Each treatment was replicated three times, with three chestnut seedlings per replicate. After collection, all samples were rapidly frozen with liquid nitrogen and stored at −80°C before further use.

Primers for the gene family members were set according to **Supplementary Table S1**, and Chinese chestnut actin was used as the endogenous reference gene. qRT-PCR was performed using a TB Green Premix Ex Taq kit (TaKaRa, Dalian). The relative expression levels of the *CmDof* gene at different time points were calculated using the 2^-ΔΔCt^ method (Livak and Schmittge, 2001). The instrument settings were: 95°C for 300 s; 40 PCR cycles, with each cycle set at 95°C for 10 s and 60°C for 30 s. The specific primer information is shown in **Supplementary Table S1**, where the Actin gene of Chinese chestnut is used as the reference gene.

## 3 Results

### 3.1 Identification and characterization of *Dof* gene family members in Chinese chestnut

We searched the whole genome data of the Chinese chestnut and obtained the hidden Markov model file (PF02701) of the Dof family from the Pfam (http://pfam.xfam.org/) database. Similar *Dof* gene sequences were obtained from the Pfam and transcriptome databases. MEGA7.0 was used for comparison. After removing the redundant sequences, 25 *CmDof* genes were identified. According to the positions of the genes on the chromosomes, they were named *CmDof1–CmDof25*. The results of sequence analysis indicate that the predicted protein sequences encoded by *CmDof*s varied significantly, with lengths ranging from 167 (CmDof9) to 530 (CmDof25) aa, corresponding to molecular weights of 18.68 kD to 57.87 kD. The theoretical isoelectric points (pI) ranged from 4.45 (CmDof3) to 9.65 (CmDof17). The instability index ranged from 40.07 (CmDof2) to 68.08 (CmDof6), with an average of 52.36. Protein hydrophobicity analysis revealed that the average hydrophobicity values were between −0.388 (CmDof17) and −1.101(CmDof7). This indicated that all CmDofs are hydrophilic proteins; however, there are certain differences in hydrophilicity exis. The strongest hydrophilicity was observed for CmDof7, and the weakest for CmDof17. Evidently, most CmDof proteins are unstable and hydrophilic. The subcellular localization of the proteins predicted that all CmDofs were located in the nucleus (**Table 1**).

**Table 1:** Basic characteristics of the putative proteins encoded by *CmDof*s.

Gene ID	Gene name	Number of amino acid	Molecular weight	Theoretical pI	Instability index	Aliphatic index	Grand average of hydropathicity	Subcellular localization
EVM0006350	CmDof1	494	53242.98	6.51	55.11	51.21	-0.821	nucleus
EVM0012965	CmDof2	286	31386.69	6.89	40.07	58.95	-0.642	nucleus
EVM0027683	CmDof3	282	31696.03	4.45	62.91	65.6	-0.631	nucleus
EVM0001652	CmDof4	189	19835.14	9.35	48.7	63.02	-0.404	nucleus
EVM0004988	CmDof5	231	24945.22	8.22	47.33	42.73	-0.833	nucleus
EVM0002949	CmDof6	282	31456.67	9.46	68.08	50.6	-0.894	nucleus
EVM0002304	CmDof7	318	35128.36	8.71	63.91	48.52	-1.016	nucleus
EVM0029581	CmDof8	358	39013.02	8.58	50.86	58.32	-0.682	nucleus
EVM0018182	CmDof9	167	18689.34	9.23	48.83	62.46	-0.596	nucleus
EVM0021532	CmDof10	491	54155.55	5.79	52.33	62.93	-0.576	nucleus
EVM0005995	CmDof11	478	52089.69	6.76	52.31	61.26	-0.699	nucleus
EVM0003112	CmDof12	279	30581.97	9.36	40.48	51.76	-0.774	nucleus
EVM0025891	CmDof13	342	36240.23	8.94	56.46	53.89	-0.597	nucleus
EVM0005005	CmDof14	294	32371.87	6.58	55.14	48.44	-0.822	nucleus
EVM0033091	CmDof15	319	34584.52	9.2	58.55	60.25	-0.626	nucleus
EVM0024156	CmDof16	328	35525.31	8.52	47.03	54.15	-0.702	nucleus
EVM0010675	CmDof17	250	26506.94	9.65	54.41	73.36	-0.388	nucleus
EVM0022839	CmDof18	234	24150.58	8.51	43.89	45.85	-0.528	nucleus
EVM0028640	CmDof19	326	36083.46	7.72	56.3	52.67	-0.898	nucleus
EVM0014602	CmDof20	509	56472.59	8.32	52.96	59.21	-0.738	nucleus
EVM0011581	CmDof21	317	34945.94	6.35	44.11	55.36	-0.686	nucleus
EVM0003878	CmDof22	284	31118.47	9.13	54.83	45.35	-0.827	nucleus
EVM0026422	CmDof23	349	36528.46	9.22	59.52	51.55	-0.532	nucleus
EVM0033450	CmDof24	272	30209.64	6.7	43.77	59.12	-0.627	nucleus
EVM0015584	CmDof25	530	57879.07	6.64	51.12	58.47	-0.561	nucleus

### 3.2 Phylogenetic analysis of CmDofs

**Figure 1:**
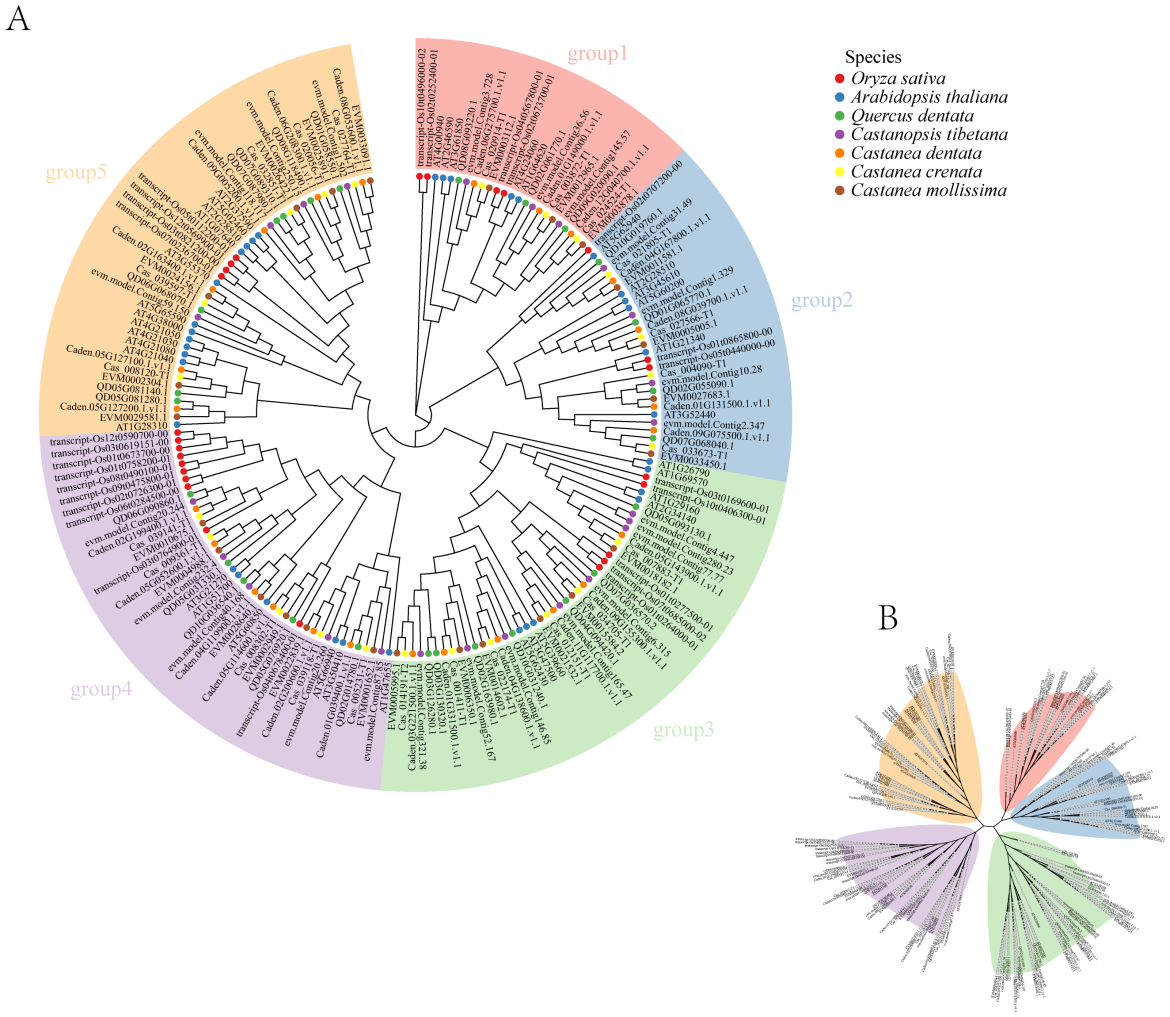
Phylogenetic tree based on the amino acid sequences of different species. Different branch colors in the figure indicate different groups; different colors of gene names indicate different orthogroups. **(A)** Phylogenetic tree based on the amino acid sequences of different species. **(B)** Phylogenetic tree based on the amino acid sequences of Chinese chestnut.

To explore the phylogenetic relationship of Dof transcription factors in Chinese chestnuts, a phylogenetic tree was constructed using the amino acid sequences of Dof from chestnuts. We observed that the 25 Dof members could be divided into five groups(Group 1, Group 2, Group 3, Group 4 and Group 5) (**Figure 1B**). Group 1 was consisted of three members, Group 2 of four members, and the remaining groups (Groups 3−5) of six members. To gain a deeper understanding of the evolutionary relationships of the Dof family among different species, sequences from several species, such as *Arabidopsis thaliana*(36), *Oryza sativa*(26), and *Quercus dentata*(26), *Castanopsis tibetana*(24),*Castanea dentata*(25), *Castanea crenata*(23),and *Castanea mollissima*(25) were collected to construct a phylogenetic tree and further clarify the evolutionary relationship of the *Dof* gene family in Chinese chestnut. The study revealed that all species could be classified into five evolutionary groups (Groups 1–5), with Groups 1 and 2 having relatively fewer genes (**Figure 1A**). *Arabidopsis thaliana* genes were enriched in Group 5 (12 genes), whereas *Oryza sativa* genes were mainly concentrated in Group 4 (10 genes). *Castanea* species generally had more genes in Group 3 (6–8 genes) (**Supplementary Table S2**). This classification result was consistent with that of the *Castanea dentata*. Groups 1 and 2 contained three and four genes, respectively, in several Chinese chestnut species. The number of genes in these two groups was the lowest in *Arabidopsis thaliana* and *Oryza sativa*, indicating that they are relatively conserved.

### 3.3 Chromosome localization and correlation analysis of *CmDof*s gene in Chinese chestnut

**Figure 2:**
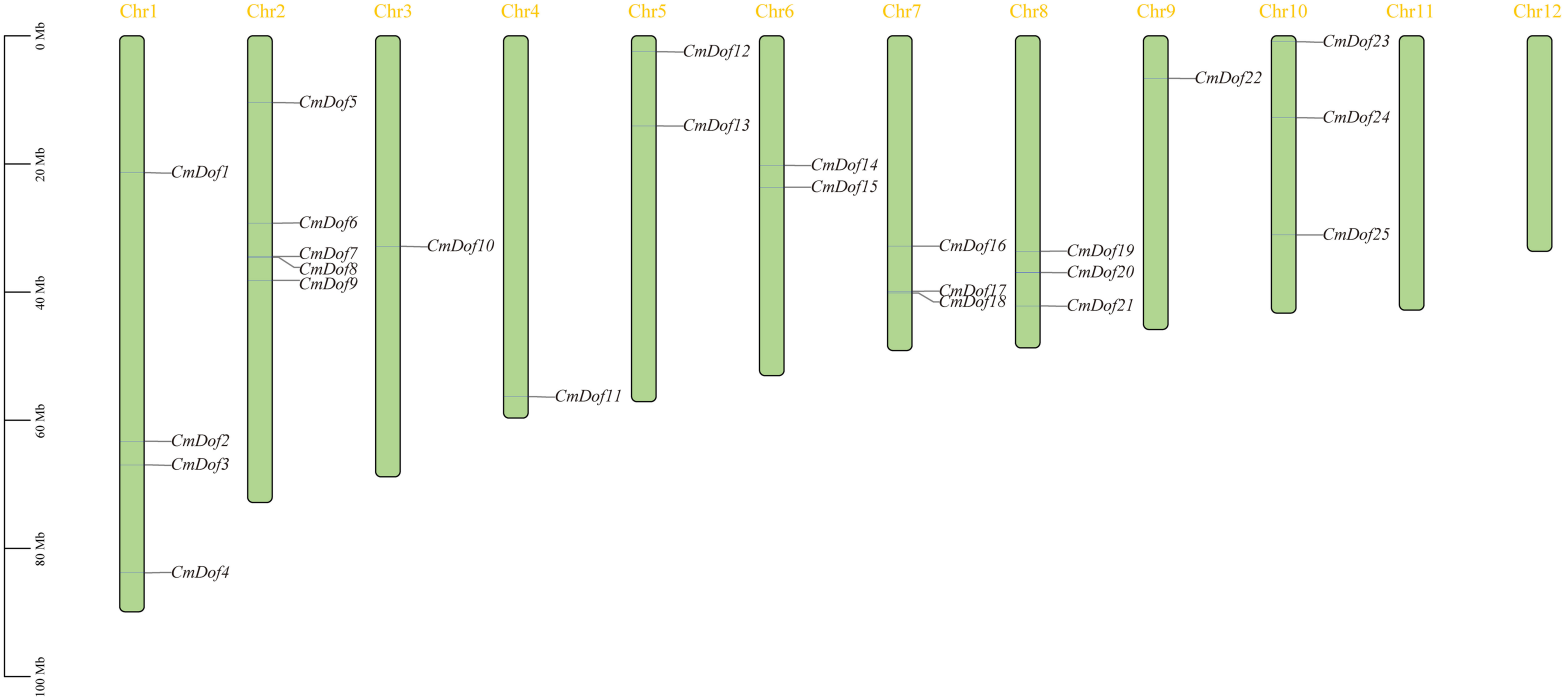
The istribution of the *CmDof* genes on the Chinese chestnut chromosomes. Vertical colored bars represent the chromosomes of Chinese chestnut. The gene name and number are shown at the right/left of each chromosome. The scale bar on the left represents the length of the chromosomes.

Our aim was to explore the location of *CmDof*s genes on chromosomes. As shown in **Figure 2**, the 25 *CmDof*s of Chinese chestnut are unevenly distributed across 10 chromosomes of Chinese chestnut, with none on chromosomes 11 and 12. Chromosomes Chr3 (*CmDof10*), Chr4 (*CmDof11*), and Chr9 (*CmDof22*) contained one gene each, while Chr5 (*CmDof12* and *CmDof13*) and Chr6 (*CmDof14* and *CmDof15*) contained two genes each. The remaining 18 *Dof* genes, accounting for 72% of the total genes, were located on the other five chromosomes. Chromosomes Chr2, Chr5, and Chr9 formed gene clusters containing a minimum of three genes each; chromosome Chr2 had the largest number of genes, with five genes (**Figure 2**).

**Figure 3:**
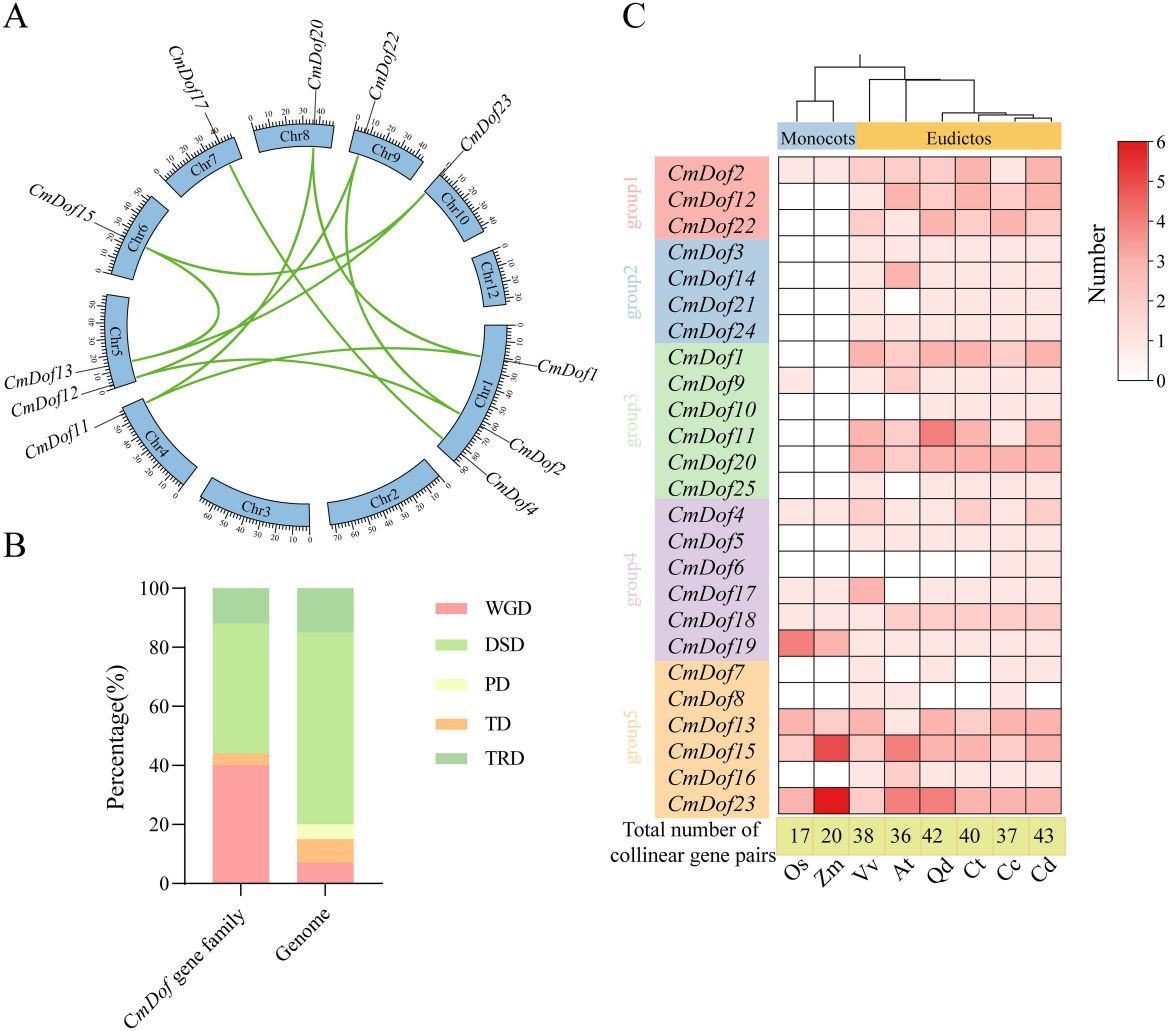
Analysis of collinearity and duplication types in the *CmDof* genefamily. **(A)** Intra-genomic collinearity of the *CmDof* genes. **(B)** Duplication types of the *CmDof* genes. TRD, DSD, PD, TD and WGD indicate transposon duplication, dispersed duplication, proximal duplication, tandem duplication and whole genome duplication in the figure. The same below **(C)** The number of genetic combinations of Chinese chestnut with other species.

To explore the evolutionary patterns, divergence and selection pressure of homologous *CmDof* genes, *CmDof*s, OsOFPs and AtOFPs were used for further analysis. By conducting a collinearity analysis of all genes within the Chinese chestnut genome, nine pairs of collinear genes were identified(**Figure 3A**). Based on the collinearity results, analysis of the gene replication types revealed that the proximal duplication (PD) replication type was completely lost compared to the entire Chinese chestnut genome, whereas the translocation duplication (TRD), tandem duplication (TD), and whole genome duplication (WGD) replication types increased significantly in the *Dof* gene family(**Figure 3B**). Further Ka/Ks calculations were conducted, and the results showed that the Ka/Ks values of the nine pairs of genes were all less than 0.3 (**Supplementary Table S3**). Further collinearity analysis was performed between the Chinese chestnut and other species, and a heatmap was drawn. The smallest number of collinear gene pairs was found with rice (17 pairs), whereas the largest number was observed with Chinese chestnut (43 pairs). The heatmap shows that all members of Group 2 were only co-linear with dicotyledonous plants, suggesting that these *CmDof* members were formed after the differentiation of monocotyledonous and dicotyledonous plants. Genes such as *CmDof13*, *CmDof15*, and *CmDof23* had collinear members in all species, suggesting that they may play an important role in plant adaptation to environments (**Figure 3C**).

### 3.4 Gene structure, conserved motif analysis

**Figure 4:**
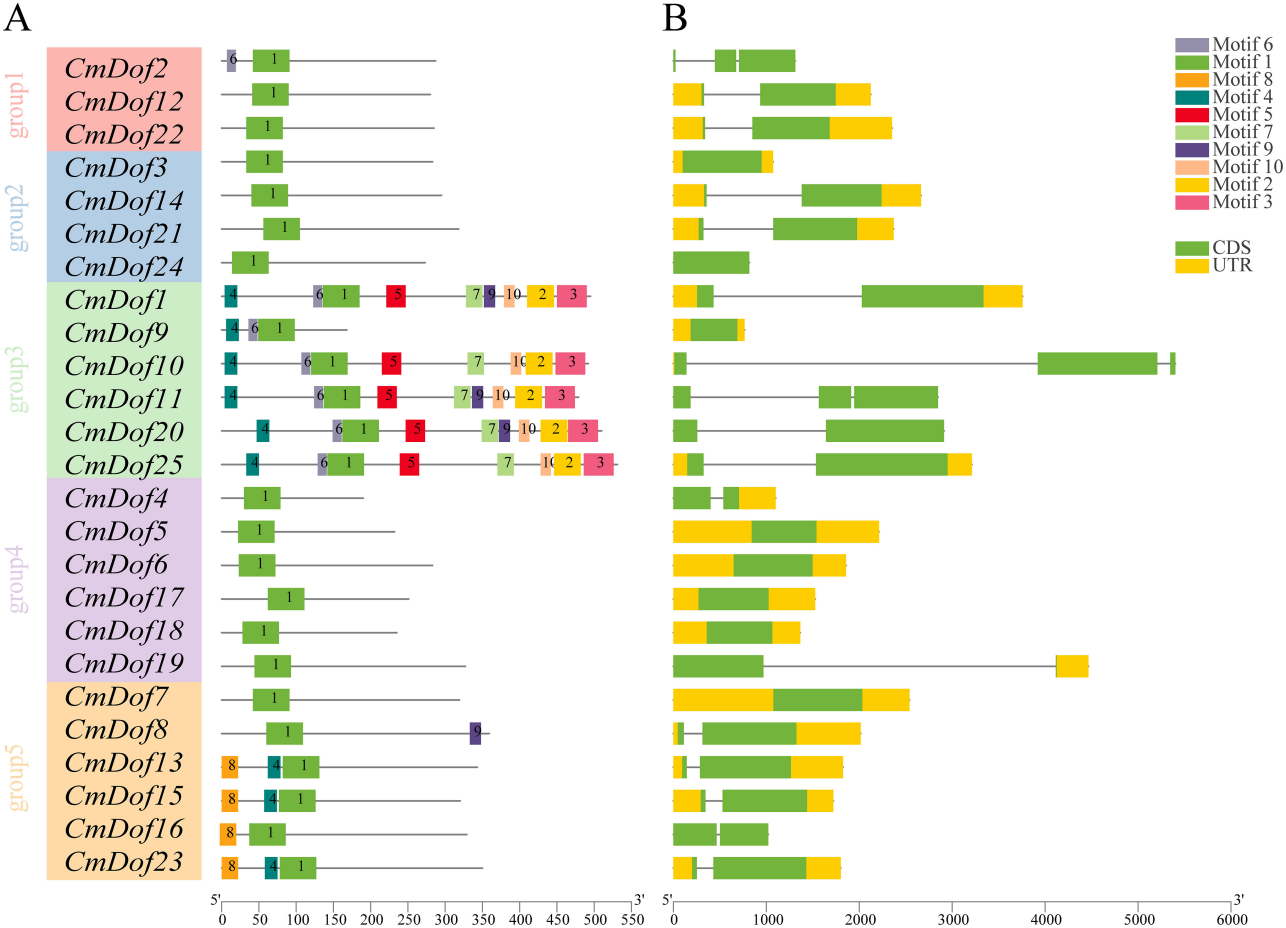
**(A)** The ten conserved motifs in CmDofs. Conserved motifs of the CmDofs were identified using the online MEME program based on 25 full-length amino acid sequences with the following parameters: maximum number of motifs, 10; maximum width, 100. The lengths and positions of different motifs in the protein sequences are identified by the lengths and positions of the different color blocks. **(B)** Gene structure of *CmDof*s. Exons, introns, and untranslated regions (UTRs) are indicated by green rectangles, black lines, and yellow rectangles, respectively.

We identified ten conserved motifs, ranging in length from 13 amino acids (motif 6) to 51 amino acids (motif 1), to characterize the structural features of the 25 CmDof proteins. Motif 1 is distributed in almost all CmDof proteins and is the Dof zinc finger domain. The distribution of the other nine motifs exhibited branch specificity, as shown in the phylogenetic tree in **Figure 4**. Motifs 2, 3, 5, 7, and 10 were only present in Group 3. Motif 8 was found only in Group 5 (**Figure 4A**). These sequence analysis results are consistent with the classification of the evolutionary tree.

We compared the DNA sequences of *CmDof* genes and examined the organization of exons and introns outside the open reading frames to investigate the evolution of *CmDof* genes in Chinese chestnut. The number of introns in *CmDof* generally varied from 0 to 2. In summary, 8 genes, accounting for 32% of the total genes, had no introns, whereas 14 genes had one intron (**Figure 4B**).

### 3.5 *Cis*-acting element analysis in promoters of *CmDof*s

**Figure 5:**
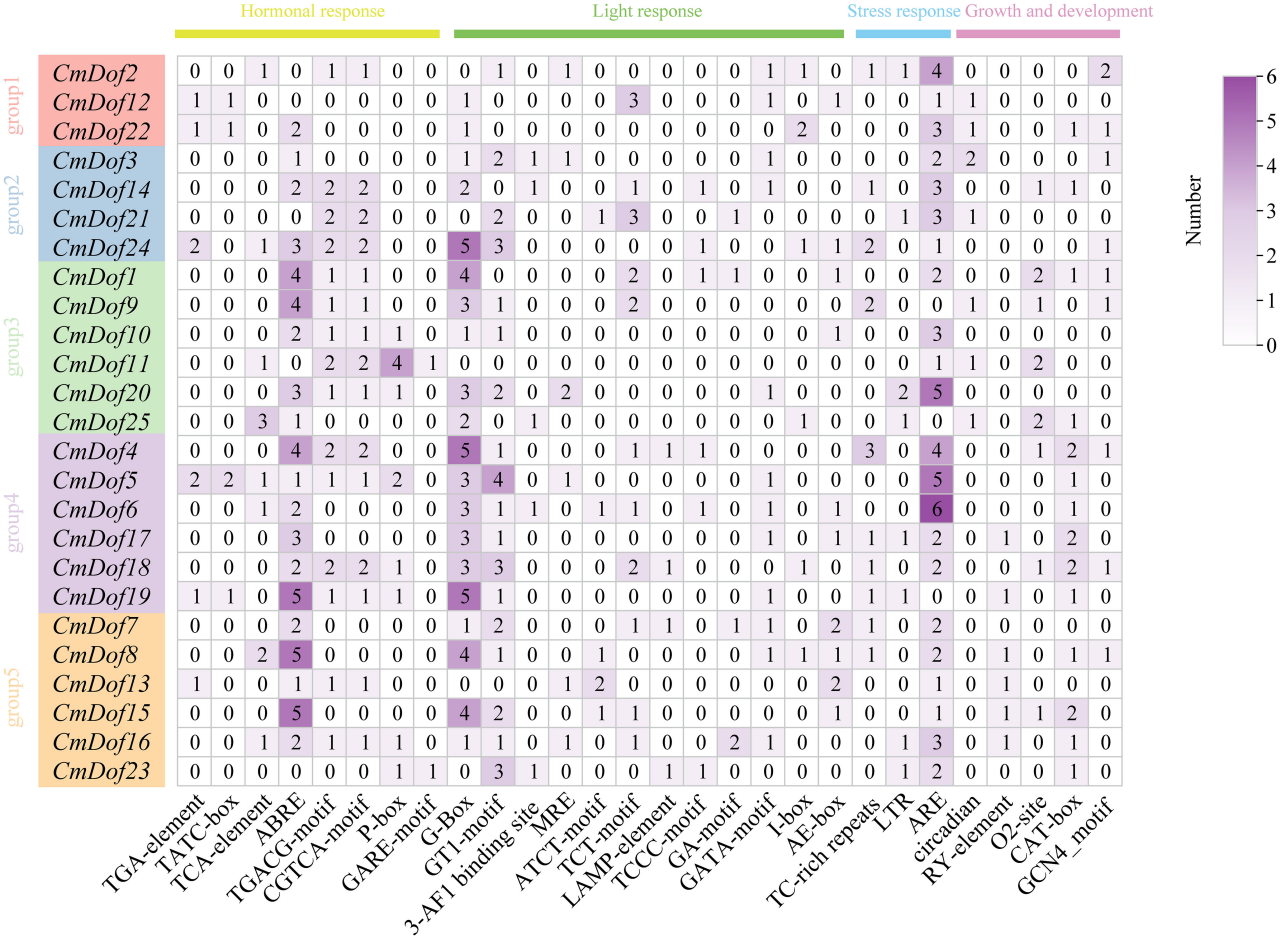
*Cis-* elements in the promoter regions of *CmDofs.* The colored block with a number represents the cis-element number of *CmDofs*.

To explore the *cis*-element patterns and types in the promoter of *CmDof*s, the 2,000 bp sequences of upstream promoter regions were used for further analysis on PlantCARE. Analysis of the promoter sequences indicated that the Chinese chestnut *Dof* gene family is rich in elements related to rapid response, photosynthetic reaction, and stress response. The light-responsive element (G-box) The light-responsive element (G-box) was the most abundant, accounting for 45.7% of all cis-elements and showing enrichment in *CmDof4*, *CmDof7*, and *CmDof19.* Hormone-responsive elements, including ABRE (ABA) and TGA-box (auxin), were most densely distributed in Group 3. Stress-related elements, such as TC-rich repeats (defense) and MBS (drought), were concentrated in *CmDof12* and *CmDof22*. Notably, the G-box element was present in 20 *CmDof* genes, with *CmDof4*, *CmDof19*, and *CmDof24* each containing five copies (**Figure 5**).

### 3.6 Analysis of gene expression patterns

**Figure 6:**
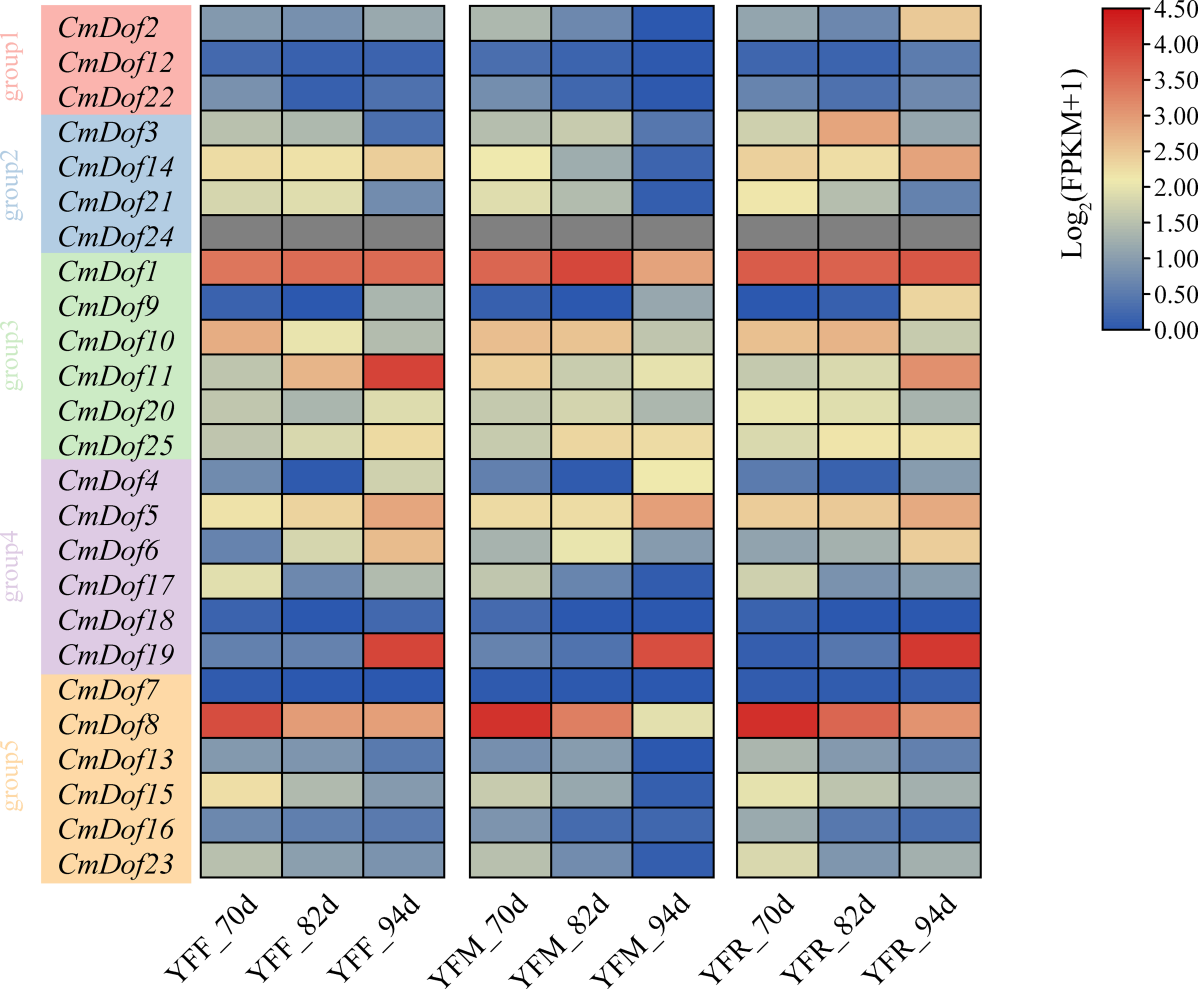
The heatmap shows the expression pattern of *CmDof* genes. YFF,YFM, and YFR indicate seeds from crosses of ‘Yongfeng 1’×’Yongfeng1,’ ‘Yongfeng1’×’Yimen1,’and’Yongfeng1’×’YongrenZao,’ respectively.70d,82d,and94d indicate70,82,and 94 days after pollination,respectively. The Roman numerals along the right-hand side of the figure indicate log_2_FPKM.

Based on the published transcriptome data from the reference genome version N11-1, we calculated the fragments per kilobase of transcript per million mapped reads (FPKM) values to analyze gene expression patterns. Among the three chestnut varieties, the expression profiles of *Dof* transcription factors were highly similar. All *CmDof* genes were clustered into five subgroups based on their expression profiles. Group 1 had almost all members with extremely low expression levels (FPKM < 0.5) in each sample. Similarly, in Group 5, except for *CmDof8*, the expression levels of the other 5 *CmDof*s were also very low. In contrast, in Group 3, the expression levels of the *CmDof*s were all relatively high, especially *CmDof1*, which had the highest expression level. This suggests that Group 3 may play a relatively important role in the process of fruit development (**Figure 6**).

### 3.7 Expression analysis under different stress treatments

**Figure 7:**
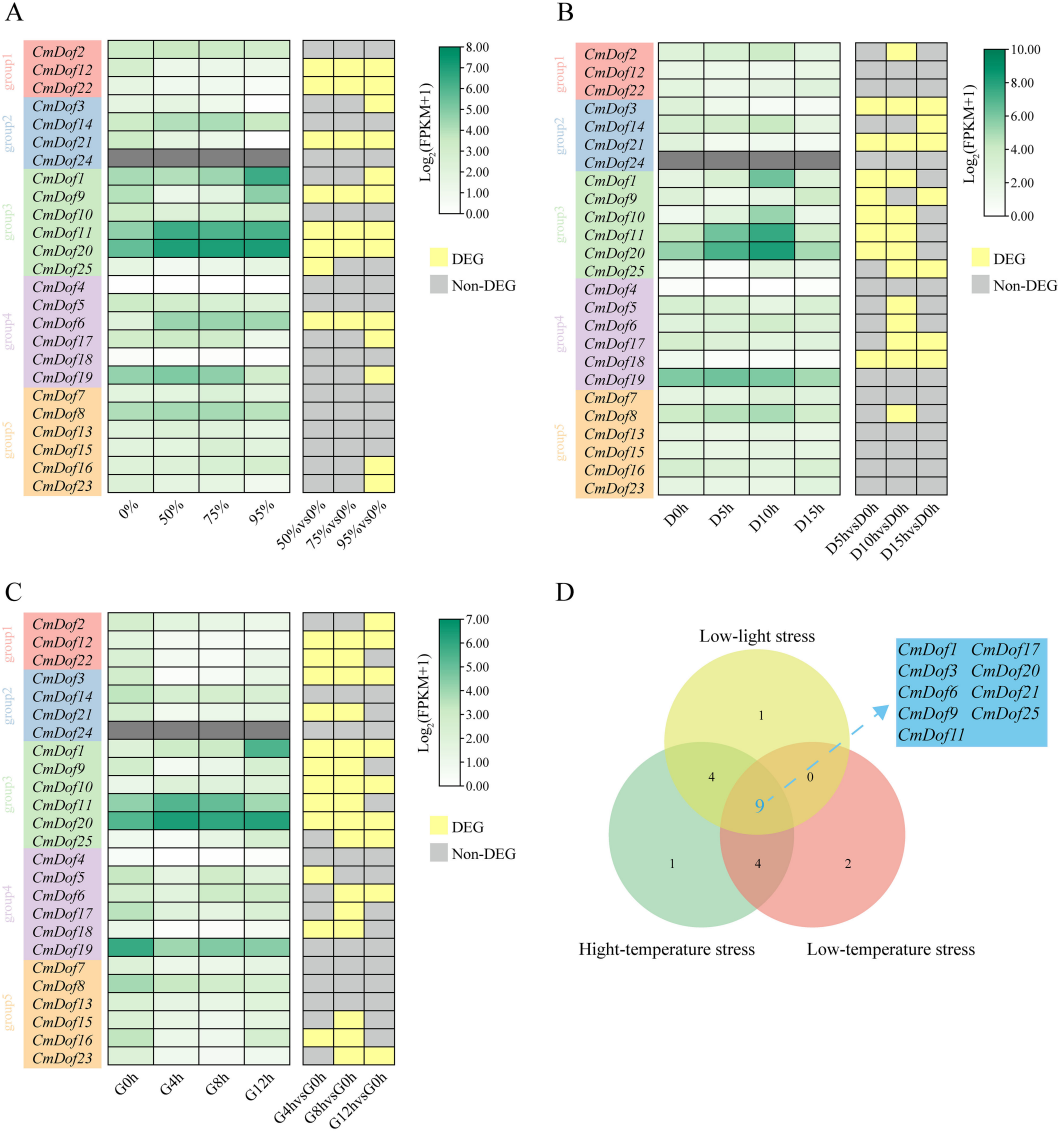
RT-qPCR of *CmDof* genes. **(A-C)** RT-qPCR of *CmDof* genes in *C.mollissima* leaves under Shade,cold and heat stresses,respectively. **(D)** Venn diagram of differentially expressed genes under three types of stress treatments.

Fragments of each FPKM per thousand base transcripts were determined to explore the expression pattern of the *CmDof* gene based on published transcriptome data under three stress treatments (shading, low-temperature, and high-temperature) (**Figure 7A–C**). Group 3 showed significant expression differences under the three treatments, while Group 1 did not. Group 5 showed no significant differences in expression under shading and low-temperature treatments but showed significant differences under the high-temperature treatment. A Venn diagram shows that the nine *CmDof* genes showed significant differences in expression under the three treatments (**Figure 7D**). Therefore, these *CmDof*s were verified by RT-qPCR. The results reveal that the expression levels of the nine *CmDof*s differed significantly. *Dof1*, *Dof6*, *Dof11*, *Dof20*, and *Dof21* were significantly increased under the three stress treatments, whereas *Dof3* and *Dof17* were significantly decreased. The expression levels of *Dof9* were increased under shading and low-temperature stress and decreased under high-temperature stress. *Dof25* increased under shading and high-temperature stress and decreased under low-temperature stress (**Supplementary Figure S1**).

## 4 Discussion

### 4.1 Identification, structural and phylogenetic analysis, and evolutionary characterization of *CmDof*s

The first *Dof* gene identified in plants was the *ZmDof* gene in maize. Since then, many Dof proteins have been identified in various plants, including rice (Khan et al., 2021), eggplant (Wei et al., 2018), pigeonpea (Malviya et al., 2015), grape (Wang et al., 2021), banana (Dong et al., 2016), apple, tea (Yu et al., 2020), durian (Khaksar et al., 2019), cherry (Hou et al., 2024), pear (Liu et al., 2020a), and pitaya (Alam et al., 2024a). The number of Dof genes identified in the complete genomes of different plants varies; there are 36 *Dof* family genes in the genome of thale cress, 30 in rice, 46 in maize, and 96 in wheat. In this study, 25 *Dof* genes were identified in the *CmDof* gene family. Its quantity is significantly lower than that of other species. These findings suggest that the *Dof* gene family in chestnut may have undergone gene loss during evolution, indicating potential evolutionary divergence among different plant species.

Systematic classification has important implications for CmDof analysis. Phylogenetic analysis revealed that the *CmDof* gene family is divided into five evolutionary branches (Groups 1–5) (**Figure 1**). The distribution of genes in each branch differed significantly (**Supplementary Table S2**). Among the *Dof* genes of *Arabidopsis thaliana*, the number is the highest in Group 5 (12 genes), while the *Dof* genes of rice (*Oryza sativa*) are mainly concentrated in Group 4 (10 genes), and the Chinese chestnut has the largest number of genes in Group 3 (6–8 genes). This suggests that the functions of *Dof* genes vary among species.

Gene structure and motif distribution can serve as supporting evidence for the evolutionary relationships between species or genes (Riechmann et al., 2000; Qu and Zhu, 2006). In general, members of the same subfamily have similar exon/intron structures and motif distribution patterns, indicating their functional similarity. However, the distribution of the number of introns in the taxonomic subgroups of Chinese chestnut did not follow this pattern exactly. This result is consistent with that of Lotus (Cao et al., 2022). The Dof gene of Chinese chestnut contains very few introns (zero to two introns), which is similar to Arabidopsis, tea, and cassava *Dof* genes (Yu et al., 2020; Zou et al., 2019). However, Chlamydomonas, Physcomitrella patens, Selaginella moellendorffii, and Pinus taeda contain four, six, five, and four introns, respectively (Moreno et al., 2007). The results revealed that intron loss occurred during the evolution of the Chinese chestnut. In addition, previous studies of gene families have found that genes with no introns and intron deficiency (three or fewer) are more likely to play a role in abiotic stress responses, such as drought and salt, than intron-rich genes (Liu et al., 2021). However, further experiments are needed to analyze the specific functions of poor intron family genes in plant growth, development, and resistance to abiotic stress. The *CmDof* gene family can be used as a resource for a poor-intron gene family, which provides important information for exploring the origin, evolution, and function of plants.

Multiple sequence alignment was used to compare the amino acid sequences in the Dof structural domain of Chinese chestnuts. The CmDofs structural domain sequences were found to be highly similar, with all containing motif1. Furthermore, the conserved structure of *CmDof* genes was similar to that of rice (Lijavetzky et al., 2003), sorghum (Kushwaha et al., 2011), and wheat (Liu et al., 2020b), suggesting that the structure of *Dof* genes is highly conserved in different species.

### 4.2 Expression profiling of *CmDof*s

Transcription factors are proteins that regulate the expression of downstream target genes by binding to specific promoter regions (*cis*-acting elements), thereby regulating protein translation during physiological processes (Li et al., 2021a). *Cis*-acting elements are essential for gene expression (Liu et al., 2014), and gene promoter investigation is crucial for understanding the general control of gene expression in plants (Hernandez-Garcia and Finer, 2014). Analysis of *cis*-acting elements revealed that the promoter regions of *CmDof* genes contain elements related to light regulation, plant growth and development, hormone responsiveness, and stress responsiveness, suggesting their roles in adapting to environmental changes during growth and development.

After the Dof protein was identified in maize, the roles of the Dof transcription factor family in plant growth and development have also been reported, including responses to non-biological stress, promotion of seed germination, induction of plant flowering, promotion of plant nitrogen assimilation, enhancement of plant photosynthesis, and increase in protein accumulation in seeds (De Paolis et al., 1996; Venkatesh and Park, 2015). The expression levels of most *Dof* genes in tea plants (*Camellia sinensis*) change when exposed to cold, heat, salt, and drought stress (Li et al., 2016); the *Dof* genes in bamboo are widely involved in cold, salt, and drought stress responses; the *Dof* genes in poplar are involved in ABA responses (Wang et al., 2017); *TaDof14* and *TaDof15* are significantly upregulated in wheat (*Triticum aestivum*) under drought treatment (Shaw et al., 2009); the five tomato (*Solanum lycopersicum*) genes homologous to the *Dof* gene of *Arabidopsi*s (SlCDF1-5) are induced to express under cold, salt, osmotic, and heat stress (Corrales et al., 2014); and most *StDof* genes are induced by drought, salt, and ABA non-biological stress in potatoes (*Solanum tuberosum*) (Venkatesh and Park, 2015). Furthermore, we found that nine *CmDof* genes showed significant responses under the three stress treatments (high temperature, low temperature, and shade). This result is similar to those of the pitaya and betel palm (Alam et al., 2024a, 2024). Moreover, the promoters of these genes contained *cis*-acting elements related to stress.

The evidence supporting the involvement of *CmDof* genes in abiotic stress responses, based solely on transcript level changes, remains preliminary. To further elucidate their functions, future studies will employ functional validation approaches such as heterologous expression in model systems. The genome editing technologies have already been applied to improve various crop traits (Alam et al., 2024b) and are being explored in chestnut to enhance specific genetic features related to stress resistance and productivity.

## Data Availability

The original contributions presented in the study are included in the article/**Supplementary Material**. Further inquiries can be directed to the corresponding author/s.

## Supplementary material

Supplementary Figure 1 Expression analysis of 9 *CmDof*s under shading, high temperature, and low temperature stress conditions.
