# Supplementary figures and images for: Genome-wide identification of DNA-binding with one finger transcription factor genes in Chinese chestnut and their response to abiotic stress
Source: Front Plant Sci. 2025 Dec 4;16:1711429. doi: 10.3389/fpls.2025.1711429 (PMC12711724; doi:10.3389/fpls.2025.1711429)

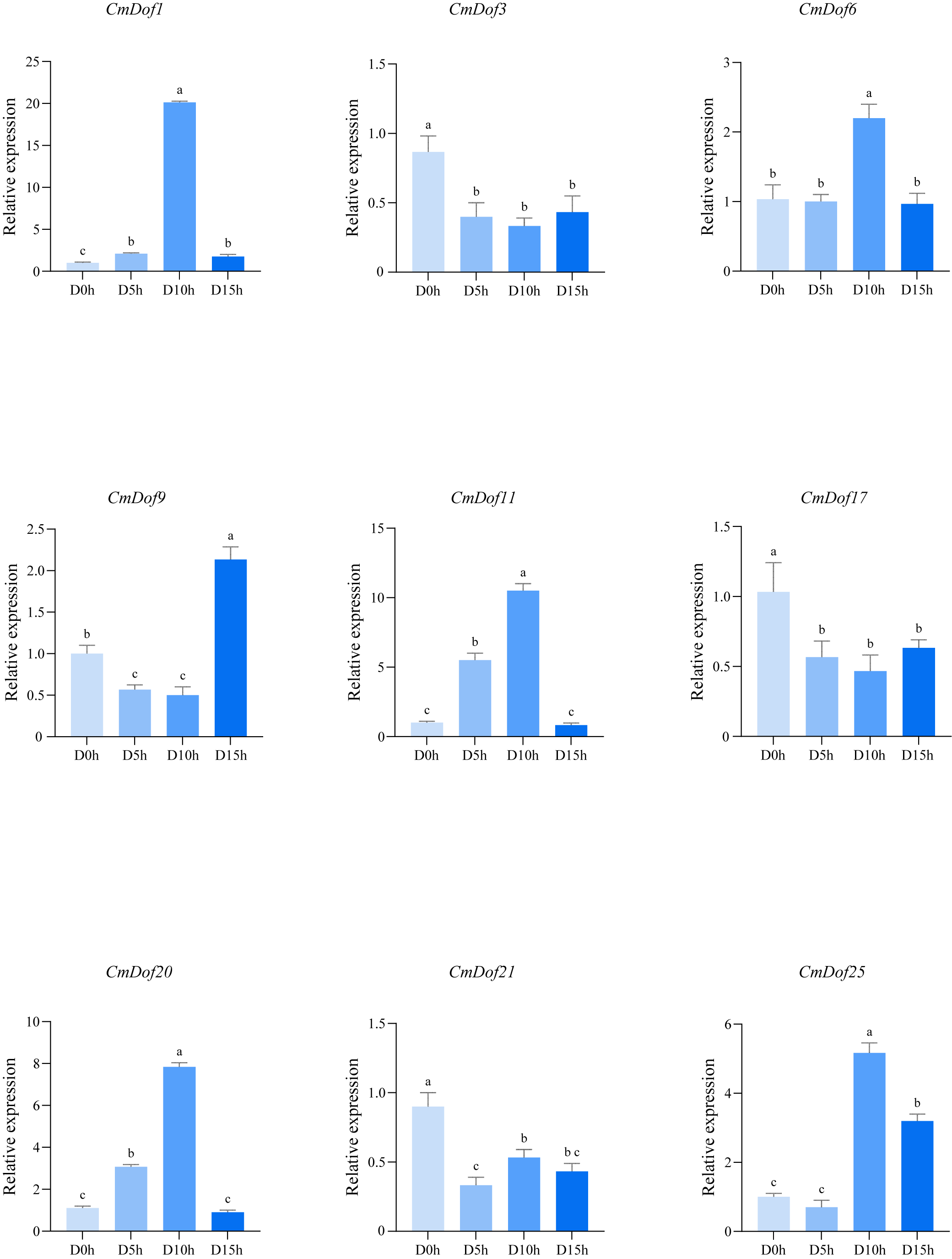

Supplement: Supplementary Figure 1 — Expression analysis of 9 CmDofs under shading, high temperature, and low temperature stress conditions. [file Image1.tif]

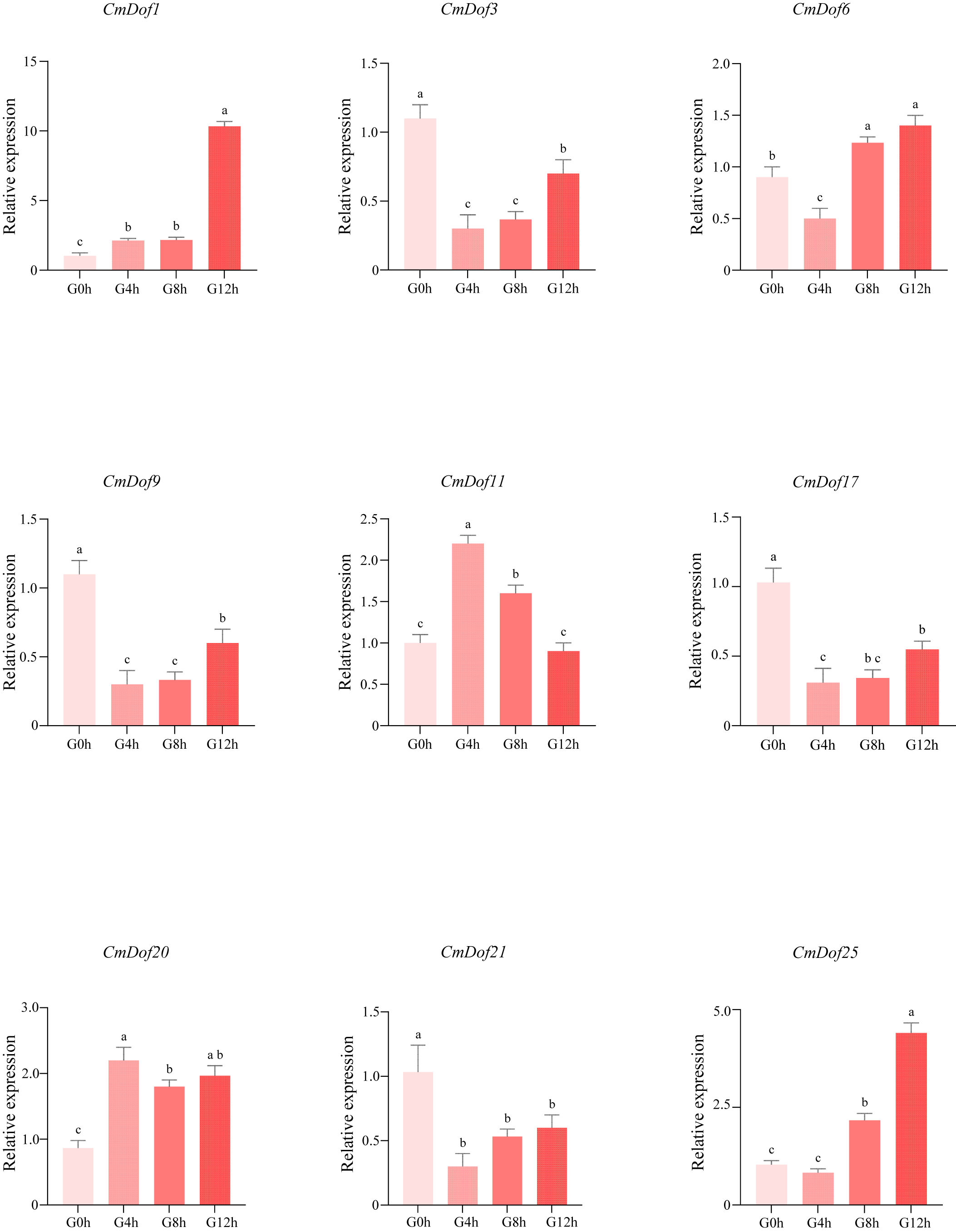

Supplement: Supplementary file 2 [file Image2.tif]

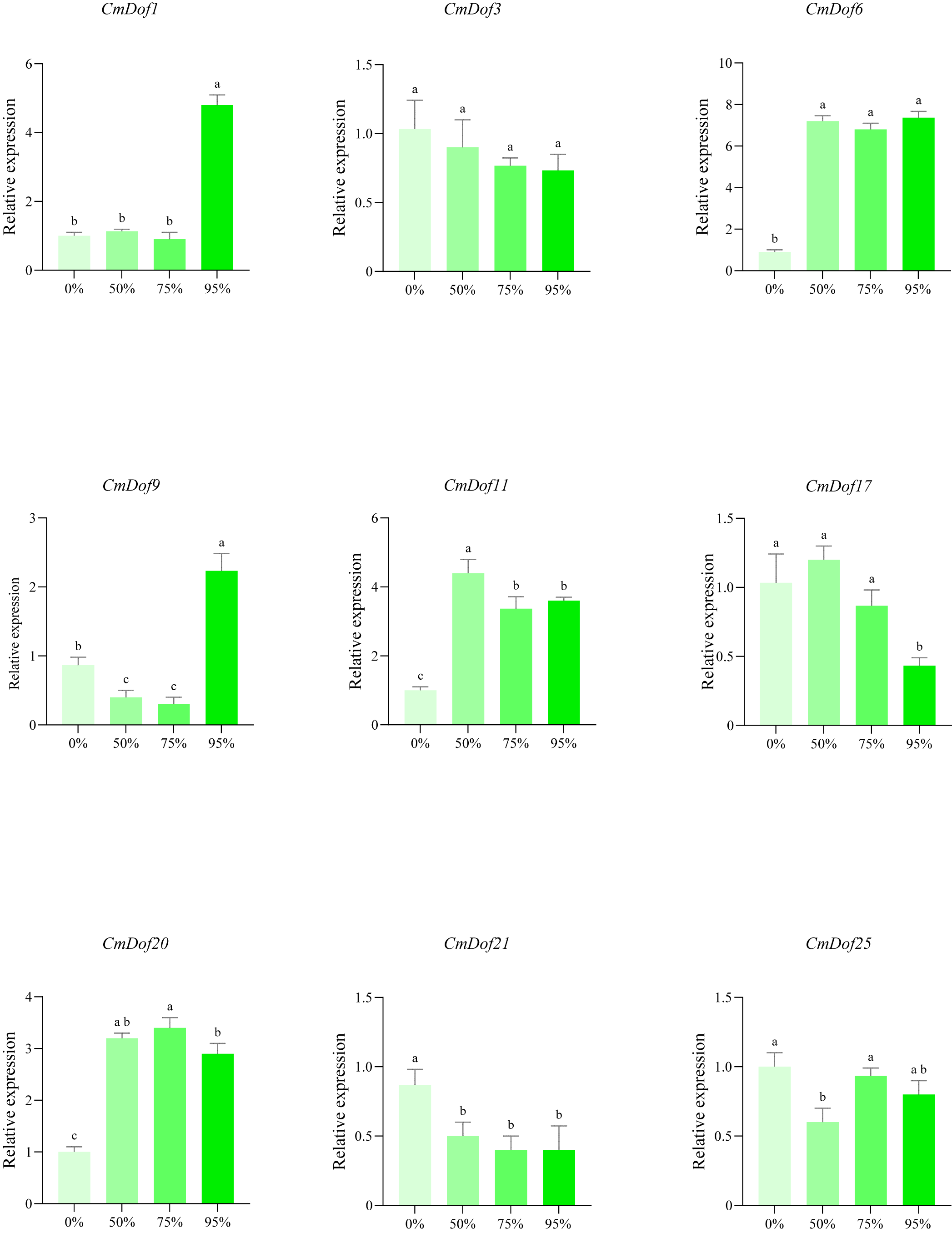

Supplement: Supplementary file 3 [file Image3.tif]
