# Supplementary material for: Genome-wide identification of DNA-binding with one finger transcription factor genes in Chinese chestnut and their response to abiotic stress
Source: Front Plant Sci. 2025 Dec 4;16:1711429. doi: 10.3389/fpls.2025.1711429 (PMC12711724; doi:10.3389/fpls.2025.1711429)
Supplement: Supplementary file 4 [file Table1.docx]

**Table S1**.:Primer sequences for qRT-PCR.

|  | Sequence ID | Primer ID | Primer Sequence |
| --- | --- | --- | --- |
| *CmDof1* | EVM0006350.1 | EVM0006350.1_1_F | CCATGCCCCCGCTGTAAT |
|  | EVM0006350.1 | EVM0006350.1_1_R | TGCAGAAATGCCGGGGTT |
| *CmDof2* | EVM0012965.1 | EVM0012965.1_1_F | TGCCCATGCCAGTTCAGG |
|  | EVM0012965.1 | EVM0012965.1_1_R | CCAACTCCAAGCCCATCCA |
| *CmDof3* | EVM0027683.1 | EVM0027683.1_1_F | GCCCTCGTTGTGCTTCCT |
|  | EVM0027683.1 | EVM0027683.1_1_R | GAGCCACCTTTGGTCCAGT |
| *CmDof4* | EVM0001652.1 | EVM0001652.1_1_F | CCACTGCTACCAACGGCA |
|  | EVM0001652.1 | EVM0001652.1_1_R | CCGCCAAAGTCACCGACT |
| *CmDof5* | EVM0004988.1 | EVM0004988.1_1_F | CCTTTCACAGCCTCGCCA |
|  | EVM0004988.1 | EVM0004988.1_1_R | CGGCTTCCACCACCAACT |
| *CmDof6* | EVM0002949.1 | EVM0002949.1_1_F | CAAGTCACAGCCTCGGCA |
|  | EVM0002949.1 | EVM0002949.1_1_R | CGCCACCCACAGGAACAT |
| *CmDof7* | EVM0002304.1 | EVM0002304.1_1_F | ACACCACAACCACCGCAA |
|  | EVM0002304.1 | EVM0002304.1_1_R | ACGAACGTGGGCCATACC |
| *CmDof8* | EVM0029581.1 | EVM0029581.1_1_F | GCTCTCAGGTGTCCTCGC |
|  | EVM0029581.1 | EVM0029581.1_1_R | TGTCCAGTACCGCTTGCA |
| *CmDof9* | EVM0018182.1 | EVM0018182.1_1_F | GACGCAAAACCAAGCCGG |
|  | EVM0018182.1 | EVM0018182.1_1_R | TGCAACCCAGAAGCAGCA |
| *CmDof10* | EVM0021532.1 | EVM0021532.1_1_F | TGGCTCTTCCTTGGCTGC |
|  | EVM0021532.1 | EVM0021532.1_1_R | CCGCAATGTCCAGGCAGA |
| *CmDof11* | EVM0005995.1 | EVM0005995.1_1_F | GCAATGCCTCCACCTGCT |
|  | EVM0005995.1 | EVM0005995.1_1_R | GCTTTGGCTACCCTGGCT |
| *CmDof12* | EVM0003112.1 | EVM0003112.1_1_F | TGCTCAATGGCCAAAGGGT |
|  | EVM0003112.1 | EVM0003112.1_1_R | GCTCCTGAGGCCTTGCTC |
| *CmDof13* | EVM0025891.1 | EVM0025891.1_1_F | TCCACCACCACAGTTGCC |
|  | EVM0025891.1 | EVM0025891.1_1_R | CACCACTACCACCGCCTG |
| *CmDof14* | EVM0005005.1 | EVM0005005.1_1_F | CATGTGGGCAGTTGGGGT |
|  | EVM0005005.1 | EVM0005005.1_1_R | GCCAGGACCAGGGTTTCC |
| *CmDof15* | EVM0033091.1 | EVM0033091.1_1_F | TCAACCCCGTCACTTCTGC |
|  | EVM0033091.1 | EVM0033091.1_1_R | CGGCAACCTCCACCAACT |
| *CmDof16* | EVM0024156.1 | EVM0024156.1_1_F | TGCCCTCGTTGTGACTCAC |
|  | EVM0024156.1 | EVM0024156.1_1_R | ACGGCAACCACCACCAAT |
| *CmDof17* | EVM0010675.1 | EVM0010675.1_1_F | GGTGGTGCCCTCCGAAAT |
|  | EVM0010675.1 | EVM0010675.1_1_R | ATCGAAGCCGGTTCCGAG |
| *CmDof18* | EVM0022839.1 | EVM0022839.1_1_F | TTTGGGCTTGGGAGAGCG |
|  | EVM0022839.1 | EVM0022839.1_1_R | CTGCCACGTGTTCCCCAT |
| *CmDof19* | EVM0028640.1 | EVM0028640.1_1_F | CAACAAACACCGCCACCG |
|  | EVM0028640.1 | EVM0028640.1_1_R | CCGAGCCTTGTTCCAGCA |
| *CmDof20* | EVM0014602.1 | EVM0014602.1_1_F | CCTGCCCTCGCTGCAATA |
|  | EVM0014602.1 | EVM0014602.1_1_R | GCAGAAATGGCGTGGCTG |
| *CmDof21* | EVM0011581.1 | EVM0011581.1_1_F | CCACCGCATGACCAAGCT |
|  | EVM0011581.1 | EVM0011581.1_1_R | GGGTCCCACCTTTGGTCC |
| *CmDof22* | EVM0003878.1 | EVM0003878.1_1_F | TCCACCAGCTCATGAGGACT |
|  | EVM0003878.1 | EVM0003878.1_1_R | TGGTGGCTTTTGCTGTTGC |
| *CmDof23* | EVM0026422.1 | EVM0026422.1_1_F | CTTCCACCTCCACCACCG |
|  | EVM0026422.1 | EVM0026422.1_1_R | GCCATGGATCCAGGCCTG |
| *CmDof24* | EVM0033450.1 | EVM0033450.1_1_F | TTGCCACCATTGCCAGGT |
|  | EVM0033450.1 | EVM0033450.1_1_R | ACTGGCAGCTGCGTTTCT |
| *CmDof25* | EVM0015584.2 | EVM0015584.2_1_F | TGCTTGCAGTTCCAGGCT |
|  | EVM0015584.2 | EVM0015584.2_1_R | GAGACACTCCCAGCCCCA |

**Table S2.**:The number of genes in each branch of the evolutionary tree for different species.

| Species | group1 | group2 | group3 | group4 | group5 | Total |
| --- | --- | --- | --- | --- | --- | --- |
| *Oryza sativa* | 4 | 3 | 5 | 10 | 4 | 26 |
| *Arabidopsis thaliana* | 5 | 6 | 7 | 6 | 12 | 36 |
| *Quercus dentata* | 3 | 4 | 7 | 5 | 7 | 26 |
| *Castanopsis tibetana* | 3 | 4 | 8 | 5 | 4 | 24 |
| *Castanea dentata* | 3 | 4 | 6 | 6 | 6 | 25 |
| *Castanea crenata* | 3 | 4 | 6 | 5 | 5 | 23 |
| *Castanea mollissima* | 3 | 4 | 6 | 6 | 6 | 25 |

**Table S3.**:The Ka, Ks, Ka/Ks Values of Gene Pairs Identified by McScanX.

| Gene pairs | | Ka | Ks | Ka/Ks |
| --- | --- | --- | --- | --- |
| *CmDof12* | *CmDof22* | 0.25 | 1.20 | 0.21 |
| *CmDof2* | *CmDof22* | 0.23 | 1.24 | 0.18 |
| *CmDof11* | *CmDof1* | 0.25 | 1.22 | 0.20 |
| *CmDof12* | *CmDof2* | 0.26 | 1.13 | 0.23 |
| *CmDof11* | *CmDof20* | 0.29 | 2.03 | 0.14 |
| *CmDof1* | *CmDof20* | 0.22 | 1.39 | 0.16 |
| *CmDof23* | *CmDof13* | 0.29 | 1.43 | 0.21 |
| *CmDof13* | *CmDof15* | 0.29 | 1.91 | 0.15 |
| *CmDof23* | *CmDof15* | 0.37 | 2.84 | 0.13 |
